# Biological and Mechanical Synergies to Deal With Proton Therapy Pitfalls: Minibeams, FLASH, Arcs, and Gantryless Rooms

**DOI:** 10.3389/fonc.2020.613669

**Published:** 2021-01-21

**Authors:** Alejandro Mazal, Juan Antonio Vera Sanchez, Daniel Sanchez-Parcerisa, Jose Manuel Udias, Samuel España, Victor Sanchez-Tembleque, Luis Mario Fraile, Paloma Bragado, Alvaro Gutierrez-Uzquiza, Nuria Gordillo, Gaston Garcia, Juan Castro Novais, Juan Maria Perez Moreno, Lina Mayorga Ortiz, Amaia Ilundain Idoate, Marta Cremades Sendino, Carme Ares, Raymond Miralbell, Niek Schreuder

**Affiliations:** ^1^Centro de Protonterapia Quironsalud, Madrid, Spain; ^2^Grupo de Física Nuclear and IPARCOS, U. Complutense Madrid, CEI Moncloa, Madrid, Spain; ^3^Instituto de Investigación Sanitaria del Hospital Clínico San Carlos, Madrid, Spain; ^4^Sedecal Molecular Imaging, Madrid, Spain; ^5^Department of Biochemistry and Molecular Biology. U. Complutense, Madrid, Spain; ^6^Department of Applied Physics, U. Autonoma de Madrid, Madrid, Spain; ^7^Center for Materials Microanalysis, (CMAM), U. Autonoma de Madrid, Madrid, Spain; ^8^Leo Cancer Care, Knoxville, TN, United States

**Keywords:** proton therapy, FLASH, minibeams, arc therapy, gantry

## Abstract

Proton therapy has advantages and pitfalls comparing with photon therapy in radiation therapy. Among the limitations of protons in clinical practice we can selectively mention: uncertainties in range, lateral penumbra, deposition of higher LET outside the target, entrance dose, dose in the beam path, dose constraints in critical organs close to the target volume, organ movements and cost. In this review, we combine proposals under study to mitigate those pitfalls by using individually or in combination: (a) biological approaches of beam management in time (very high dose rate “FLASH” irradiations in the order of 100 Gy/s) and (b) modulation in space (a combination of mini-beams of millimetric extent), together with mechanical approaches such as (c) rotational techniques (optimized in partial arcs) and, in an effort to reduce cost, (d) gantry-less delivery systems. In some cases, these proposals are synergic (e.g., FLASH and minibeams), in others they are hardly compatible (mini-beam and rotation). Fixed lines have been used in pioneer centers, or for specific indications (ophthalmic, radiosurgery,…), they logically evolved to isocentric gantries. The present proposals to produce fixed lines are somewhat controversial. Rotational techniques, minibeams and FLASH in proton therapy are making their way, with an increasing degree of complexity in these three approaches, but with a high interest in the basic science and clinical communities. All of them must be proven in clinical applications.

## Introduction: Advantages and Pitfalls in Proton Therapy

Proton therapy has been evolving as the reference for conformal radiation therapy for decades and, in spite of an exponential growth, it is still limited to less than 1% of the patients treated with radiation therapy even in high-income countries. The primary advantages of proton therapy—compared with conventional photon beams—that justify its use and development are:

1. there is not a maximum of dose in the path of a beam;
2. it may have a small lateral penumbra in the path of a beam;
3. it is possible to irradiate homogeneously (or with a controlled inhomogeneity) a target in depth even with a single beam;
4. the range of particles can be placed anywhere by changing the energy of the beam;
5. there is a high gradient of dose after the range;
6. there is no practical dose beyond the distal gradient, i.e., the beam stops;
7. the radiobiological efficiency is managed in clinics with a rather low risk.

But there are also pitfalls:

1. the entrance dose can be higher than the usual with photon beams (no skin sparing), depending on parameters such as the proximity of the target volume to the skin, the thickness of the target, the delivery technique;
2. the lateral penumbra in depth, at depths close to the range and in the region of the target volume, can be higher than mid-energy photon beams (e.g., 6 MV) as such used in rotational VMAT techniques with photons;
3. there are large uncertainties on the position of the range in complex tissues including inhomogeneities, imposing large margins to get robustness of plans, and placing the higher Linear Energy Transfer (LET) beyond the target limits.
4. There are complex dose distributions and large uncertainties beyond implanted materials such as metallic screws, rods, prosthesis, …;
5. There is a neutron dose in the tissues around the target, even far from it; it has been reduced in the evolution of proton systems from passive to pencil beam, but still there, comparing with photons beams with energies lower than 10 MV;
6. It is complex to irradiate moving targets, even more when using scanned pencil beams, in spite of specific protocols of repainting and organ movement management (interplay effect, undesirable doses beyond the target,…);
7. Capital and operational costs are high, uptimes are sometimes limited and it is difficult to easily have backups in case of system failure.

**Figure 1:**
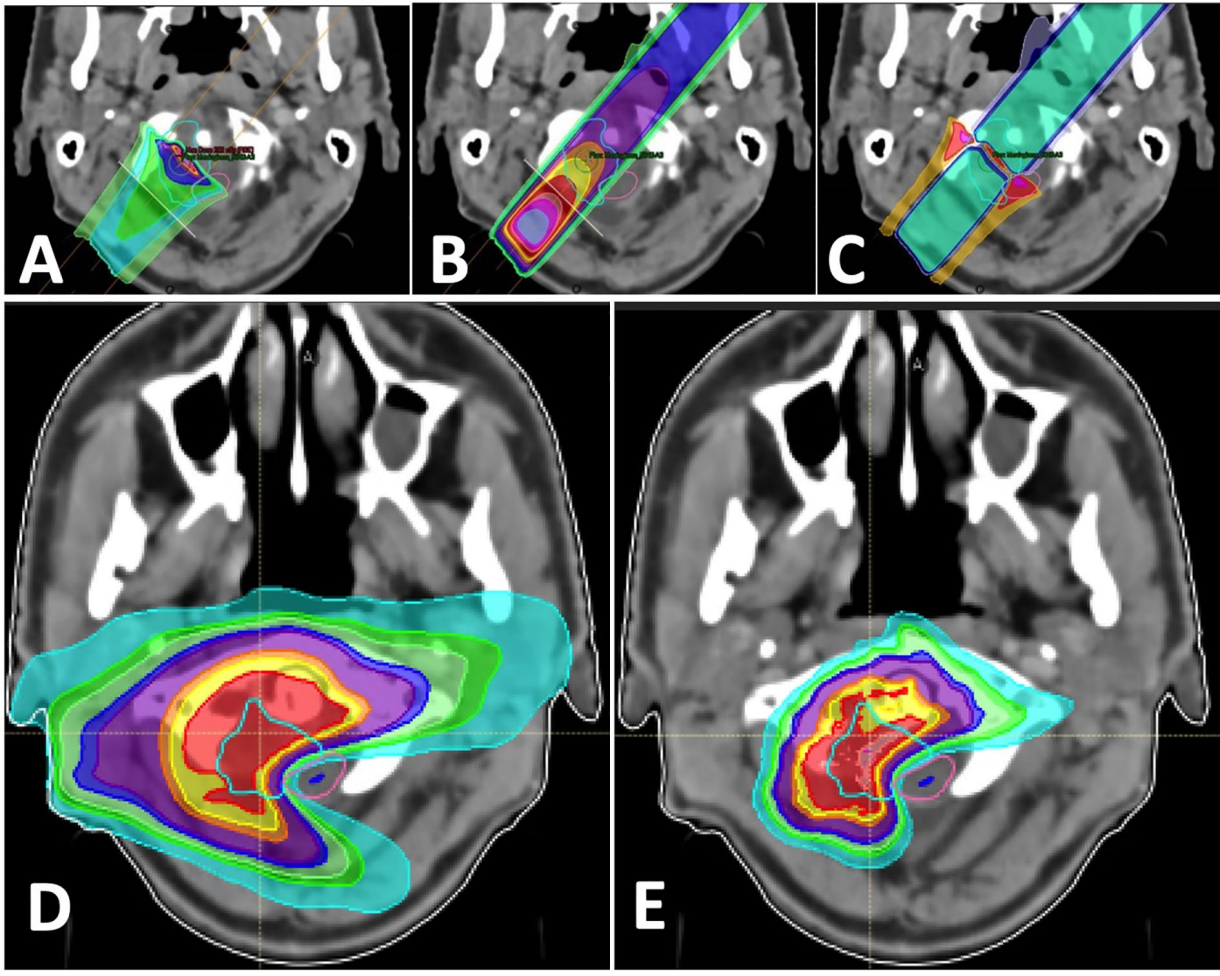
Beams for a treatment of a base of skull tumor: **(A)** single proton beam; **(B)** single photon beam; **(C)** difference between single proton (higher dose in red) and photon beams (higher dose in blue); **(D)** combination of photon arcs; **(E)** combination of proton beams.

In **Figure 1**, some of these advantages and pitfalls are presented for a single proton beam in particular compared to a single photon beam and for a final clinical dose distribution.

In practice, there are clinical cases where the dose gradient between a target and a close critical organ maybe better achieved with photon beams. But the integral dose distribution will always be easier to optimize with proton beams, given their finite range (no exit dose) that reduce the irradiation to large volumes of healthy tissues.

The advantages offered by proton therapy are stronger than the pitfalls, justifying the fact that more than 200,000 patients have been treated with protons in the world to date with more than 20,000 new treatments added per year. Proton therapy is a rational choice among the existing tools in radiation therapy for some clinical targets: pediatrics, ophthalmic, base of the skull, reirradiations. Most of the other clinical sites in radiation oncology are under investigation to quantify the real clinical advantage of the use of protons and the associated cost, through studies of tumor control, complications and quality of life. Concepts like “model-based approach” are used to evaluate individual cases, and Qualy, quality of life, cost-benefit and similar ones for a population-based study.

The use of photon beams is also still evolving, many more scholars are working actively in the photon therapy space and the pace of innovation is high. The development of any modality in radiation therapy (photons, electrons, neutrons, protons, heavy particles …) can be synergic and not opposed between them. Innovations such as the use of online magnetic resonance imaging, adaptive therapy and the combination with immunological approaches are examples of major improvements to be shared.

Several papers are included in this special issue to deal with some of the proton therapy pitfalls, trying to reduce or to eliminate them, or at least to control and mitigate their effect.

In this work we want to review and address biological and mechanical proposals to mitigate most of the mentioned pitfalls, using particular approaches to distribute the dose in space (minibeams) and time (FLASH effect) as well as to reduce complexity (rotational therapy) and cost (gantry less facilities), to make proton therapy more accessible to the benefit of more cancer patients.

## Biology: Revisiting Radiation Biology to Improve Healthy Tissue Protection

The location of the tumors and the nature of the treatments inevitably leads to a certain degree of undesired effects in surrounding tissues. Proton minibeam radiation therapy (pMBRT) and ultra-high dose rate (FLASH) radiotherapy (FLASH RT) are two innovative radiotherapy modalities where the potential to reduce normal tissue toxicity have already been demonstrated, compared to standard radiotherapy, potentially revolutionizing the radiotherapy field.

Recently several reviews on the tissue sparing and tumor control with Flash have been published, including a few oriented towards proton therapy (1). One of us and co-authors presented a review of minibeams and FLASH radiation therapy, with both approaches working independently or in synergy (2).

In this work we review some of the basic and new proposals on these subjects and how they are linked to advances in ongoing mechanical aspects of proton therapy such as proton arc therapy.

### Flash

Recent pre-clinical studies have found that the “new” methodology named “FLASH”, which consists of delivering single doses of 5 to 10 Gy in a single microsecond pulse or in times lower than 100 to 500 ms, produces a dramatic decrease of damage to normal tissues while keeping the anti-tumoral effect (3–6). This FLASH effect was described as early as the 1970s for intestinal tumors and skin lesions. One of the pioneers, J. Hendry, who, in the 1970s and 1980s, related the amount of oxygen with the radioprotection of tissues using high intensity pulsed electron beams, recently rediscussed the clinical potential application of FLASH and, finally supported the development of proton experiments while recommending to take care having a long follow up and a better understanding of parameters and effects (7, 8). The robustness of the FLASH effect has recently been reproduced in various animal models, such as mice, rats, zebrafish, pigs, and cats (5) for several organs such as lung, skin, gut, and brain (3, 4, 9, 10).

To prove the toxicity limiting capability of FLASH RT, Favaudon et al., used a lung fibrosis model in mice and demonstrated that thoracic irradiation of mice with FLASH dose rates (40–60 Gy/s) reduced the induction of pulmonary fibrosis when compared with conventional dose rates (0.03 Gy/s) where 100% of the mice develop lung fibrosis (3). In this study, they also used a xenograft model of head and neck squamous carcinoma, a xenograft model of breast cancer, and a syngeneic model of lung cancer and found that in all three models FLASH RT was as efficient as conventional radiotherapy in reducing tumor growth (3).

The reduced radiotoxicity of FLASH RT has also been shown by the irradiation of the mice abdomen. In a recent work, Diffenderfer et al., showed that after acute radiation of 10-week-old C57BL/6J mice with either 15 Gy whole abdominal FLASH proton RT (789 Gy/s) or standard proton RT (0.9 0.08 Gy/s) acute cell loss and late fibrosis were decreased in the mice irradiated with FLASH proton therapy, whereas the effect on tumor growth was similar with the two irradiation modalities (10). This is in agreement with previous studies where the protective effect of FLASH RT in the gut was also observed (11). Using proton beams, Abel et al. (12) reported differences between FLASH (and FLASH with pulsed beams) vs “conventional” radiation for doses higher than 15 Gy on the thorax region of mice using several endpoints such as weight, dermatitis, lung function and lung fibrosis, as well as gender differences (female mice having better response to FLASH but no difference on mode of cell death).

Furthermore, it has also been shown in several studies that FLASH radiotherapy also has less neurotoxic effects compared to conventional RT (9, 13–15). Montay-Gruel et al., reported that mice with whole brain irradiated with FLASH RT experienced better preservation of memory and performed better in the behavioral studies compared to those irradiated with standard RT (9, 13).

The biological mechanism responsible for the reduced tissue toxicity following FLASH RT is yet to be fully explained. The reduced adverse long-term effects of FLASH irradiation observed in normal tissues compared to conventional dose rates and or tumor tissues have been explained by the different type and/or amount of the induced DNA damage. *In vitro* experiments suggested that the genomic instability induced in response to FLASH RT was much lower than at conventional dose rates (16, 17).

In addition to the DNA damage, several hypotheses have been proposed to explain the FLASH effect, such as the presence of free radicals or oxygen depletion that will trigger different biological responses depending on the status and metabolism of the cell (18). Oxygen depletion has been proposed to cause transient hypoxia and radio-resistance, and this is considered as the underlying mechanism, but *in vitro* data to support this assumption has been lacking until recently (19). To test the role of oxygen in the FLASH effect, Adrian et al., irradiated prostate cancer cells at different oxygen concentrations using either 600 Gy/s (FLASH) or 14 Gy/min (CONV) (20). Their results showed that in hypoxic conditions, cell survival increased in the cells irradiated with FLASH, while in normoxic conditions no differences were found between FLASH and conventional RT (20). A recent study by Montay-Gruel et al., proposes that oxygen depletion at ultra-high dose rates inhibits the production of reactive oxygen species (ROS) which promote radio-resistance (9). They report that increasing the local oxygen concentration reversed the protective effect of FLASH (9).

Furthermore, depletion of ROS using ROS scavengers sensitize zebrafish embryos to conventional therapy while having no effect in FLASH RT (9). The oxygen depletion hypothesis was used to explain the normal tissue radio-resistance to FLASH RT.

Besides local and transient oxygen depletion, radical-radical interaction is another hypothesized reason for the FLASH effect. FLASH irradiation results in a high local radical concentration available to interact with the DNA (21).

However, if tumors (or partial volumes of the tumors) are partially, but maybe not fully, hypoxic, how do they react with FLASH RT? The metabolic reorganization or the absence of proper antioxidant defenses, frequent in tumor cells, may accelerate the presence of irradiated induced radicals which may jeopardize tumor cell viability.

Nevertheless, more studies are necessary to validate these hypotheses experimentally for a full understanding of the biological effects induced by FLASH therapy.

The immune system and inflammation have also been proposed to play a role in FLASH RT protective effect of normal tissues. In their paper, Favaudon et al., found changes in the induction of the transforming growth factor beta (TGFb), a pro-inflammatory signal, which was reduced in FLASH irradiated mice (3). In addition, previous studies have shown an increased recruitment of T lymphocytes in tumors treated with FLASH-RT (22). Furthermore, a recent study in which they perform a genome-wide microarray analysis on mice that have been irradiated either with FLASH or conventional RT showed that immune system wide activation and maturation was downregulated in mice following FLASH RT (23). Therefore, these studies suggest that FLASH irradiation induces the response of the immune system in the irradiated tissue; however, the molecular mechanism behind this response remains to be explained.

Recently, Wardman (24) reviewed 60 years of experience with pulse radiolysis and highlighted 2 mechanistic approaches for the differentiated effect on normal versus tissue cells, i.e., the depletion of a chemical critical to the effect and/or the radical-radical reactions. Favaudon (25) also reviewed these two approaches, i.e., oxygen depletion vs radical recombinations, giving more weight to the second phenomena. He also stated that in both extremes of anoxia (or deep hypoxia) and hyperoxia there is no FLASH effect, making it important to know the oxygen pressure in the tumor and tissues to predict the effect. The group presented a chemical kinetic model supporting peroxyl radical recombination as the main effect (26) and, adding the results from Fouillade et al. (4), they conclude that part of the differential effect between tumors and healthy tissue could be related to DNA damage (dependent on oxygen and radicals) and double strand break repair protein 53BP1 for which tumors cells have a repair defect.

From the published data, we conclude that the main hypothesis explaining the FLASH benefit, is based on three main aspects, i.e., (a) a “window” of Oxygen concentration, (b) the kinetics of radicals and, (c) an intrinsic differentiation between tumor and healthy cells related to their DNA damage repair mechanisms. A correct understanding of the mechanisms behind FLASH effect may help to establish protocols aiming to decrease the harmful effects of ionizing radiation by preserving the healthy tissues surrounding the irradiated tumor while keeping the curative effect. A first clinical application has been reported (6) and new clinical trials are being approved. Furthermore the potential use of FLASH in pediatrics (e.g., in medulloblastoma) has been cited from studies in juvenile mice (27).

### Mini-Beams

Minibeam radiation therapy (MBRT) is an innovative strategy for spatially fractionated radiotherapy that consists of using a series of narrow (sub-millimetric) parallel beams to deliver the dose. This results in dose profiles consisting of a pattern of peaks and valleys.

The approach has an old rational with spatially fractionated “GRID” radiation therapy with photons using patterns of large peaks and valleys or sectors, both in the 1-cm scale, to spare skin toxicity with orthovoltage devices (28) and to shrink malignancies for advanced and palliative cases, with Co´60 and linacs (29), but not with curative intention.

The rationale behind the new approach is that the smaller the beam size is, the higher the dose tolerances of the healthy tissue appears to be, and a curative aim can still be kept. This is known as the dose-volume effect. Several studies have reported that MBRT is less neurotoxic than standard radiotherapy (30–35). The potential of the minibeams radiotherapy technique was studied in brain tumor bearing rats that were irradiated using X-ray minibeams. Deman et al., found that the survival time of irradiated glioma bearing rats was doubled when compared to untreated animals (30). This increase in glioma bearing rats’ life span was similar to the one obtained through other radiotherapy techniques. However, no brain damage was found on X-ray minibeams irradiated in healthy rats suggesting healthy tissues have a higher tolerance to submillimetric spatially fractionated beams (30). These experiments suggest that X-ray minibeams can be used in brain tumor radiotherapy.

Prezado et al. modified a small animal irradiator to be able to perform MBRT experiments. As a proof of concept experiment, they irradiated a group of rats with standard radiation while the other group received MBRT, both groups with 20 Gy mean dose and evaluated 6.5 months after radiation. They found that the standard RT group have extensive brain damage while in the MBRT group no significant brain lesions were observed (31). *In vitro* studies have shown that MBRT induces clonogenic cell death of human glioma cell lines (33). In a recent report by the same group showed that proton MBRT (pMBRT) increases the therapeutic window for high grade gliomas (34). They showed that pMBRT causes less neurotoxicity than standard proton therapy and in addition it significantly reduces tumor growth (34). This opens the possibility for even more aggressive irradiation schemes.

In a recent study by Dos Santos et al., they compare the micro- and nanodosimetric characteristics of three different MBRT modalities: proton (pMBRT), photon (xMBRT) and electron (eMBRT). They found that pMBRT was the most effective at preserving normal tissue since it caused less energy deposition and lower number of DNA breaks both in peak and valley cell nuclei (35). Furthermore, pMBRT was also the most aggressive treatment in the tumors region, as it was associated with a higher number of complex DNA breaks and higher energy deposition, and energy per event, at the cell nucleus (35).

As mentioned above several studies have reported the therapeutic interest of the MBRT at preclinical level, but the biological mechanisms responsible for the described protection of healthy tissues are not fully understood to date. Classically, the protective effect of MBRT on healthy tissues has been associated with the apparent resistance of normal tissue vasculature to MBRT (36). Furthermore, it has been proposed that the efficiency of MBRT on reducing tumor growth is related to a preferential damaging effect on the tumor vasculature (37). When applied to the brain of rodents, microbeam irradiation does not modify blood volume or vascular density (36). In fact, the endothelial cell lining of the vessels in the microbeam paths remains intact (37). However, immature blood vessels are more sensitive to MBRT than mature blood vessels (38). This has led to the hypothesis that immature blood vessels in the tumor will be more sensitive to MBRT while the healthy tissue mature blood vessels will be resistant to MBRT. Several reports have shown that MBRT affects the tumor vasculature structure, nevertheless, the effect may vary depending on the tumor type. In general, MBRT induces a decrease in tumor blood vessels leading to decrease in perfusion and to tumor hypoxia (39, 40). However, in a mammary tumor model, MBRT increased pericyte numbers, suggesting a normalization of the vasculature structure and tumor oxygenation (41). Although MBRT preferentially affects the tumor vasculature structure, we shouldn’t restrict the effects of MBRT to vascular effects only.

A study of the early transcriptomic responses of normal brain and glioma tissue in rats after MBRT irradiation showed that inflammation and immunity appear to be major contributors to MBRT efficacy (42). Pathways related with natural killer cells (NK) or CD8+ T lymphocytes were particularly represented in the irradiated tissue. Furthermore, they found changes in genes such as *HMGB1*, Toll-like receptors 1, 2, 7, C-type lectins 7A and CD36 in the irradiated tissue (42). These genes can trigger activation of innate or adaptive immune cells. Therefore, their hypothesis is that biochemical changes in irradiated cells, will activate these genes which in turn will promote inflammation or an immunological response (42). This is in agreement with data from Sprung et al. that have previously reported using a genome transcriptional screening that MBRT in mouse mammal tumors induced upregulation of immunity-related genes (43). Still more *in vitro* and *in vivo* experiments where the immune response within healthy tissue and/or tumor is studied in response to MBRT are necessary to fully understand the mechanisms behind MBRT. We conclude that there are still a lot of open questions about the mechanisms of action associated with MBRT.

Although the mechanisms of action and the biological effects of both FLASH and MBRT are still under study, both radiation modalities have the potential to become paradigm-changing technologies in the radiotherapy field. They can open the door to a new approach to the delivery of curative radiotherapy and may become an effective treatment for radioresistant tumors.

### The Dose Matter: The Dosimetry of FLASH and Mini-Beams

The accurate measurement of the dose delivered in a FLASH irradiation with photons, protons or electrons is a challenging task mainly due to the high dose-rate beams employed in this radiotherapy technique. Because of this, redundant measurements are usually performed with dosimeters whose response is nearly independent of the dose-rate (44).

As in the case of conventional radiotherapy, ionization chambers may be employed to measure the absolute dose, but with some caution. For instance, it has been stated by Petersson et al. (45) that the factors that correct the raw charge collected by the dosimeter in a pulsed electron FLASH irradiation depend on the dose per pulse rather than on the dose-rate.

Faraday cups have also been employed as a dosimeter in FLASH radiotherapy. In this case, the integral charge measured is used to validate the ionization chamber measurements, as shown in different studies (10, 46).

Among the dosimeters with a response independent of the dose rate, radiochromic films are commonly employed to provide a redundant verification of the dose delivered as shown in the works of Buonanno et al. (47) and Jaccard et al. (48). Also, alanine pellets have been satisfactorily employed together with radiochromic films to perform independent dose verifications in the first clinical FLASH treatment of a human patient (6). Other dose-rate independent dosimeters that have been employed for the measurement of ultrahigh dose-rate beams are the PTW microdiamond, the LYNX 2D scintillator the TLD-100 and the Methyl Viologen (44). A comprehensive review of dosimeters for FLASH including charge-based, chemicals and luminescence detectors has been presented recently (49) with interesting figures of merit in a spider chart diagram for each of them, underlying the importance of the luminescence methods for resolution in time and additional performances on measuring Oxygen tension and LET.

Finally, some experiments have been carried out to achieve real time monitoring of FLASH irradiations. For instance, Diffenderfer et al. (10) employed a NaI gamma detector to relate the prompt gamma rays detected to the dose rate of the irradiation while Oraiqat et al. (50) have stated that an ionizing radiation acoustic imaging technique may be employed to perform real-time deep tissue dosimetry.

The dosimetry of proton minibeams radiation therapy is challenging due to the fact that it should characterize the inhomogeneous entrance spatial dose distribution as well as the homogenous part of the dose distribution. The entrance dose distribution presents marked spatial variations in the millimetric and submilimetric scale thus a high spatial resolution dosimeter should be employed. On the other hand, the homogeneous part of the beam does not present markedly spatial variations thus conventional dosimeters such as ionization chambers may be used. For this reason, a two-step protocol has been proposed by Peucelle et al. (51) in order to measure proton minibeams obtained by means of a multi-slit collimator. The first step consists in absolute dose measurements performed with a thimble ionization chamber and the second step is performed with radiochromic films to characterize the peak-to-valley dose ratios.

Radiosurgery diodes have been employed as an alternative to or together with radiochromic films for measurements in the high modulated entrance dose region. In the work of De Marzi et al. (52), the high modulated entrance region is characterized by performing measurements with a radiosurgery p-type silicon diode. Microdiamond diodes have also been proposed and evaluated in the works of Meyer et al. (53) and Farr et al. (54). Also, a microdiamond diode has been employed to characterize carbon and oxygen mini-beams in the work of Martinez-Rovira et al. (55).

Finally, some experiences have been carried out with gel dosimetry as in the work of Annabell et al. (56), where fluorescent microscopy is employed to achieve higher spatial resolution dose measurements.

### The Time Factor in Beam Delivery for FLASH: The Pulsed Structure of Clinical Beams and Its Relationship With the Kinetics of the Physicochemical Processes

While it is usual to talk about the high dose rate to achieve FLASH, it is important to understand how this dose is delivered in time, in particular the pulsed structure of the beam. In two extremes, we can mention the “continuous” irradiation provided by a cyclotron (acceleration at several MHz), and the low frequency pulses of a synchrotron (a few Hz) and synchrocyclotrons (typically 1 KHz), modified by different extraction methods. The latter is gaining industrial interest from the perspective to make compact accelerators.

**Figure 2:**
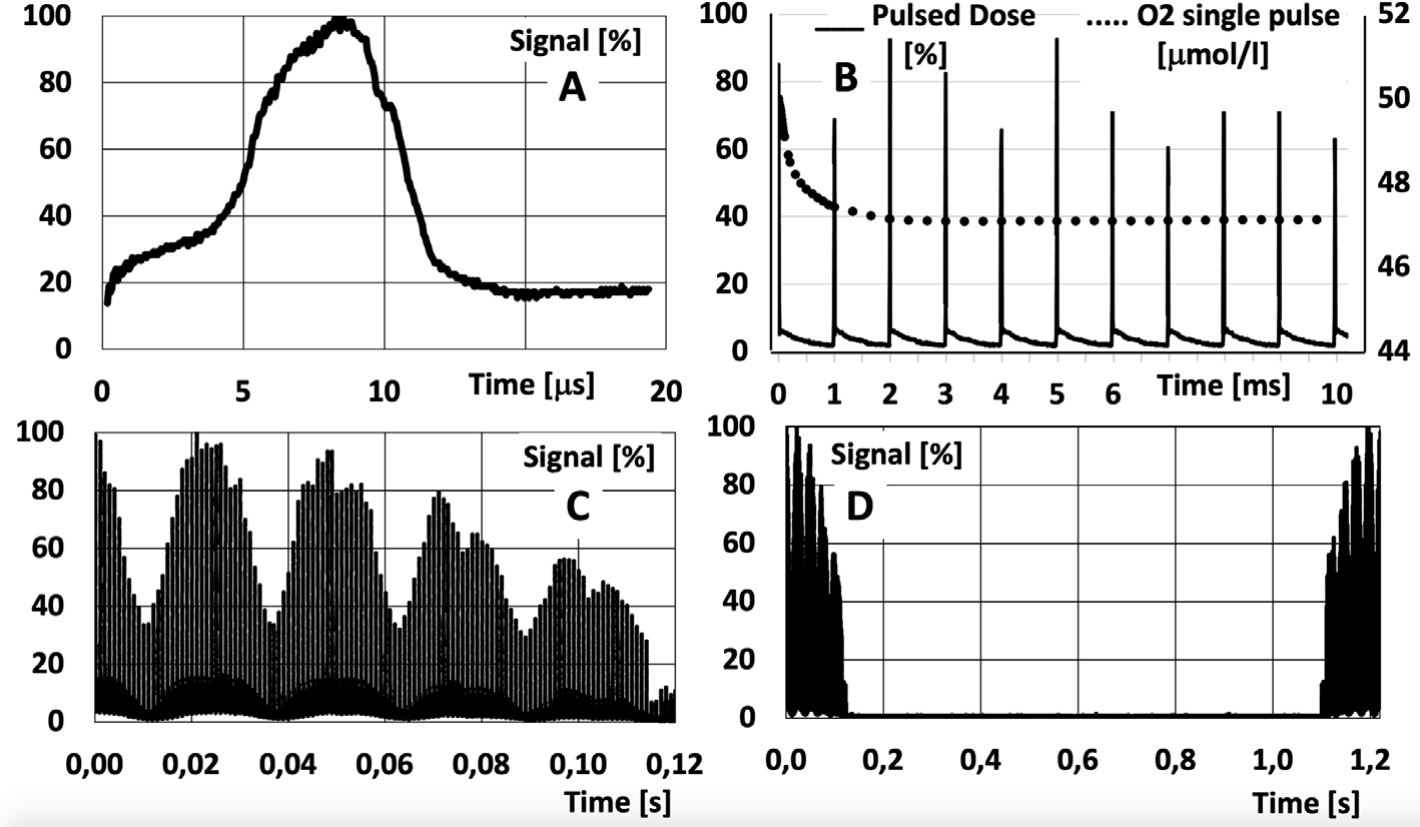
Measurement at a fixed spot in a media when delivering protons with a scanned beam from a synchrocyclotron: **(A)** single spot; **(B)** a sequence of spots delivered in a same point at an extraction frequency of 1 kHz, superposed with the effect on oxygen (O_2_) from a first pulse (see text); **(C)** effect cumulated dose in a point given by different lines scanned in a single layer (each packet is a line, the next packet is a contiguous line); **(D)** change of a layer in depth with a larger time to change energy (here about 1 sec). Measurements have been performed with a CeGAG scintillator coupled to a S13360-6075CS SiPM from Hamamatsu, read with a digital Picoscope.

Internally, a synchrocyclotron varies the frequency of acceleration to be synchronized with the particle mass when it acquires energy. Here we use the “S2C2 Proteus One synchrocyclotron” (IBA, Leuven) as an example. We measured the beam intensity arriving at the isocenter as a function of time in a clinical condition (**Figure 2**). The accelerating frequency varies from 90 to 30 MHz. At extraction, there are pulses of about 10 µs wide (**Figure 2A**) each 1 ms (**Figure 2B**), i.e., 1 KHz pulses.

In **Figure 2B**, we superposed published data (26) on the O_2_ evolution from a concentration of 50 µmol/L, as if 10 Gy were delivered at 10^6^ Gy/s in the first pulse of 10 µs wide. The potential interaction with the pulsed beam must be evaluated in any experimental study (e.g., delivering the 10 Gy in 10 or 100 pulses) for all the elements involved (Oxygen, radicals, etc.).

Other patterns of dose delivery in time must be considered. In some systems the dose deposition in a single “spot” is fractionated in two to three parts so a feedback system can measure and control the delivery of an accurate total integrated charge for the spot. At a larger scale of time, when using a “pencil beam scanning system”, the dose at a given point in the medium will have contributions from contiguous points and lines (related to scanning times) and layers (related to time to change the beam energy). In an even larger scale of time, if more than one beam is planned, several seconds or minutes are required to rotate the gantry and/or a couch to position the next beam and even more time is added if any verification of the new beam and patient position is required. The possibility to deliver FLASH in a very small number of fractions will add the scale of a daily difference between irradiations.

It is of the utmost importance to evaluate in the research programs these scales of time in particular related to the chemical and biological process mentioned before.

## Mechanics: The Advantages and Limits Of Arcs And Gantry-Less Proton Beams

Proton arc therapy is under consideration nowadays to reduce calculation complexity and uncertainties, as well as to optimize the deposition of high LET in tissues. But proton gantries are much more cumbersome and expensive than gantries for photon beams. There is a renewed interest to evaluate fixed lines and rotate the patient to reduce costs. Both approaches, arcs and fixed lines, have advantages and pitfalls we evaluate here.

### Proton Arc Therapy (PAT)

The notion of rotating the proton gantry during beam delivery, in a similar fashion as it is done for Volume Modulated Arc Therapy (VMAT) using photons, has been studied in detail by various groups in the last two decades. Except from the first study (57), which used a rotational phantom to show the favorable physical properties of protons over electrons, most studies have been limited to dosimetric calculations in patients or phantoms. These studies showed that protons arcs have indeed a better longitudinal dose profile than photons (58, 59), and that increasing the number of incoming angles could have a positive impact on the resulting dose distribution, further reducing out-of-target dose (60–62) and secondary neutron dose for passively scattered protons (63).

However, none of these studies addressed in detail the feasibility and practical aspects of the proposed solutions. Treatment planning was typically performed with standard clinical software (by simply selecting an arbitrarily large number of fields). The effect of energy layer switching time (ELST), preventing different energy layers from being delivered simultaneously, from a single control point, was not contemplated.

A group at the University of Pennsylvania (Philadelphia, PA) did publish some work on the feasibility of delivering proton arcs using passively scattered (PS) beams (64, 65) but with the global market moving inevitably towards pencil beam scanning (PBS) solutions, no new developments involving PS beams were realistic at the time. The same group also explored the feasibility of arc techniques with PBS (66), showing that, with an adequate range selection system, single- and dual-energy proton arcs (named Proton Modulated Arc Therapy, or PMAT) could achieve similar dose coverage and organ-at-risk sparing capabilities as full-coverage 2-field and 4-field intensity-modulated proton therapy (IMPT) plans (67). The same study also showed limited improvement by using fully modulated arcs, warning that existing planning systems might not be able to produce optimal proton arc therapy plans by simply combining an arbitrarily large number of field angles in an IMPT plan, and that specific treatment-planning algorithms for proton arc therapy, either developed in-house (68, 69) or as an addition to existing systems (70) are probably required.

In 2016, a research group at Beaumont Health (Royal Oak, MI) published an article describing a PAT solution named SPArc, for Spot-scaning Proton Arc Therapy (70). It is based on a patented algorithm that optimizes the number of arc control points and the number of energy layers delivered from each angle. The algorithm was implemented in Raystation (RaySearch laboratories AB, Stockholm, Sweden) and used to plan two example patient cases, showing some potential for dose reduction in healthy tissues at a cost of increased delivery and treatment planning times. These time increased by a factor of ~2 and ~10, respectively, in comparison with equivalent static IMPT plans. This team has since published several studies analyzing possible dosimetric outcomes of SPArc in various tumor sites: prostate (71), non-small cell lung cancer (72), whole brain irradiation (73), head and neck (74) and left-side breast (75). This last location has been also explored by other teams (76). **Table 1** summarizes the most relevant data from these studies.

**Table 1:** Analysis of published dosimetric studies comparing SPArc with IMPT.

Tumor site	Number of patients		Ratio of treatment times (SPArc/IMPT)*	Reference IMPT plans used clinically?	Demonstrated clinical relevance?	Reference
Prostate	9		2.0	No (VMAT)	No	(71)
Lung	14		1.2	No (IMRT)	No	(72)
Whole brain	8		0.9	No (VMAT)	No	(73)
Head and neck	14		1.1	No (combination IMPT with SFUD)**	Mean reduction of 31% in probability of salivary flow dysfunction.	(74)
Left-side breast	8		1.1	Yes	Mean reduction of 23% in probability of major coronary events, among other endpoints.	(75)

(*) Assuming a value of 1s for energy layer switching time.

(**) X. Ding, private communication. SFUD, Single-field, uniform dose.

In general, it is hard to produce convincing evidence comparing two techniques based solely on treatment planning. The physical or biological rationale supporting superiority of a technique over another must be absolutely clear: in other words, these kind of studies have to prove that not only is SPArc better than IMPT in a selection of cases, but also that IMPT could not produce equivalent results if used differently (different planning objectives, different choice of fields, etc.). Also, as is usually the case with proton therapy, it is often unclear, and not necessarily obvious, that improved dose distributions automatically imply clinically relevant improved effects. Tumor-control probability (TCP) and normal-tissue complication probability (NTCP) models are therefore useful to prove this point, waiting for real clinical trials.

All five studies published to date by the Beaumont team show a clear potential of the SPArc technique to reduce out-of-target (integral) doses, and they do so without a foreseen major impact in treatment delivery time. However, only one of them (75) clearly demonstrates clinical relevance by comparing SPArc plans and clinically used IMPT plans in terms of NTCP, showing a predicted mean reduction of 23% in the probability of major coronary events caused by a reduction in the heart dose.

Plan robustness must also be considered when discussing recent developments in proton arc therapy. The general belief is that proton arc therapy is naturally more robust than IMPT, as it spreads the range uncertainty among different beam angles (61, 77). Dosimetric studies using SPArc seem to support this hypothesis: for all reported plans in all five sites, SPArc plans present equal or better robustness than their IMPT counterparts, evaluated in terms of mean area under the curve for root-mean-square dose volume histograms for relevant organs at risk, a metric introduced by Liu et al. (78).

Another interesting effect linked with PAT is radiobiological optimization (77). Increasing the number of beam angles allows for reducing the dose delivered by high-energy beams at the distal end of the target potentially placing the high-LET components close to a critical organ. The team at the University of Pennsylvania recently showed that PMAT plans effectively increase relative biological effectiveness (RBE) within the target (68). This finding was validated with an in-vitro study (79) and has also been reported by other authors in simpler PAT implementations (80). The clinical relevance of this radiobiological effect of PAT is yet to be established.

The aforementioned potential benefits of proton arc therapy, particularly in its SPArc implementation, instigated the development of a prototype system. A patent from U. of Maryland described in 2018 a method to deliver a proton beam while the gantry rotates around the patient, without changing the energy from the source but using an automatic energy modulator (81). In 2019, the first delivery of SPArc plans was reported by the Beaumont team (82) in their IBA Proteus One accelerator, with a technique that was named Proton Dynamic Arc Delivery, or PDAD. The delivered plans reported passing all quality assurance tests (flatness, symmetry, isocentricity), and the system was able to deliver a clinical plan over a 220-degree arc in 4 min.

Further work is required before SPArc (or any other implementation of PAT) becomes clinically available. The Beaumont team (82) cite machine stability (beam pauses, interlocks) and clinical workflow (development of DICOM standard, integration with TPS and Oncology Information System, QA program) as the main issues that need to be resolved. This should be complemented by an improvement in treatment calculation time, since the current status of the SPArc dose calculation algorithm, with over 2 h per patient (70), would hinder its incorporation into a clinical workflow. While recent developments in the SPArc dose optimization algorithm (83) have reported some advances, including a ~50% reduction in estimated irradiation time, a recent study (84) has identified several inherent weaknesses in the SPArc algorithm and proposed an alternative approach which can possibly reduce planning time by up to a factor of 10.

In conclusion, while PAT does not have the disruptive aura of other advanced technologies (such as FLASH or minibeams), it can indeed produce a positive effect in the quality of IMPT plans (due to better dose conformity, increased RBE and enhanced robustness). However, this effect must be backed up by more clinical studies. It could improve the logistics of proton treatments, like VMAT with photons, provided that fast and accurate treatment planning algorithms are developed. While its integration with other novel technologies (such as FLASH or minibeams) has not been studied in detail yet, arc strategies (in the form of arc-shoot-through techniques) have been proposed as an intermediate solution for achieving FLASH dose rates with pencil-beam scanned proton beams  (85).

### Gantries vs Fixed Beam Treatment Rooms—The Need for a Change in Paradigm Enabling Treating Patients in an Upright Orientation

In a recent paper Bortfeld et al. emphasized the need for particle beam therapy to become more available to more patients (86, 87). One of the three aspects that they list to “democratize” protons is to reduce the costs of proton systems by doing away with expensive gantry systems and adopt fixed beam treatment rooms attaining multiple beam angles by rotating the patient in the beam. Fixed horizontal beams have been exploited in early systems to treat the patients in a seated position (88, 89) and at Fermi Lab, patients were treated in an upright position with neutrons (90, 91). Seated and upright treatments were until recently regarded as suboptimal arrangements forced on proton therapy when it was only available at physics research institutions, i.e., before the very large, expensive gantry systems were developed for and installed at hospital based and free-standing proton therapy clinics.

When the neutron therapy clinical results struggled to live up to the promises in the early eighties, people in the field reasoned that it is because they could not achieve the same conformality in dose than what was possible with gantry-based photon systems. This was mainly due to the lack of neutron gantries and not having multileaf collimators to allow for beam shaping to conform the beam to the target. That argument led to the development of isocentric neutron gantries and neutron multileaf collimators.

However, the proton depth dose curve (Bragg Peak) allows for a different paradigm in delivering the dose to a target. The fact that the beam stops and that fewer beams are typically used in proton treatment plans, defeats for some people the argument that proton gantries are essential. Furthermore, it is true that it’s better to treat the patient in the same position as what the patient was scanned in mainly due to the displacement of organs when the patient is moved from a lying into an upright position. Intracranial lesions can be, and have been, treated in an upright position although the patient was scanned in a lying position. Multimodality imaging is an important aspect of treatment planning and target delineation and the best registration between different modality images is obtained with the patient in the same orientation. Like CT scanners most other imaging systems, i.e., PET, MRI, PET-CT, gamma Cameras and even Ultrasound scans are often designed to image the patient in a lying position. This notion further supports the thinking that radiation therapy treatments should be done in the lying position.

**Figure 3:**
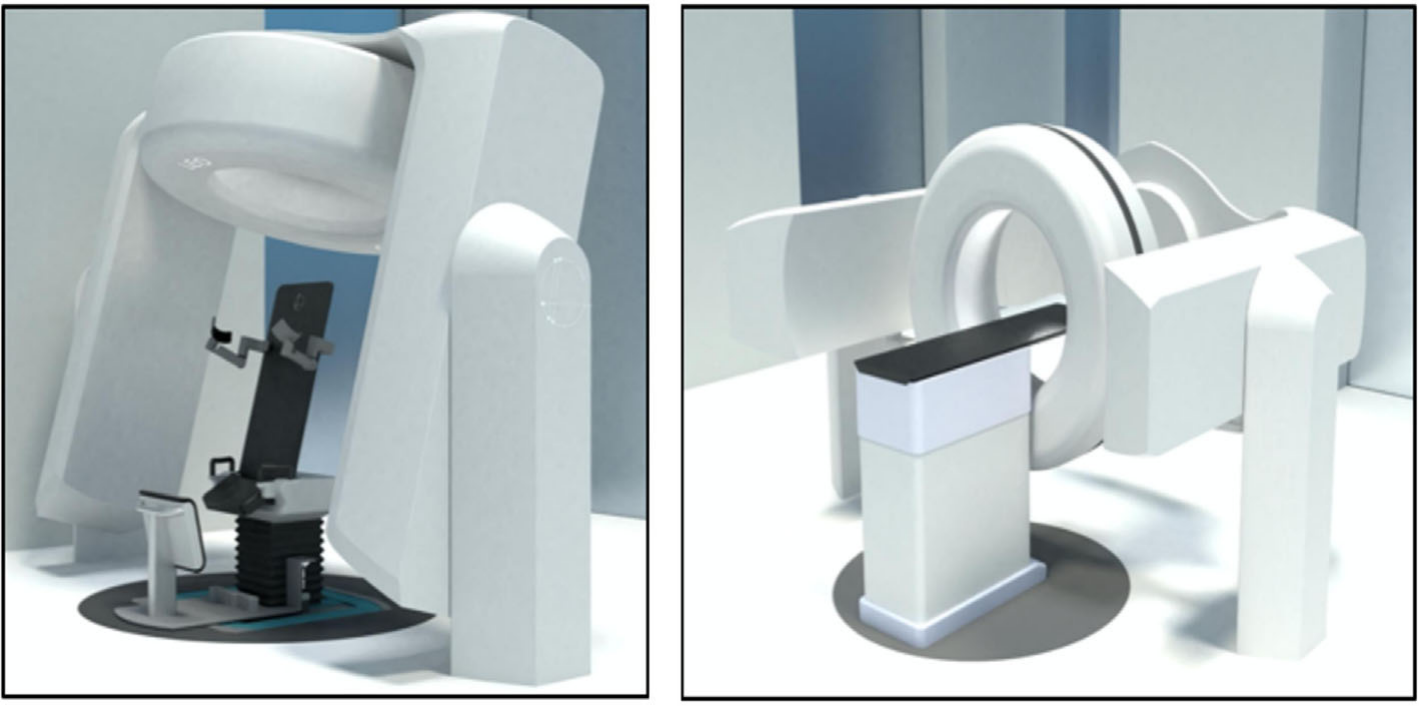
The left panel shows an upright Dual Energy CT scanner together with the upright patient positioner for upright scanning and treatments that is under development. The right panels show the CT scanner integrated with a CT gurney for CT scanning in the lying down position (courtesy LEO Cancer Care Ltd).

This paradigm is shifting, and several companies are now developing technologies that will allow for imaging and treating patients in an upright orientation (companies such as P-Cure, New-RT Corp Ltd, LEO and Advanced Oncotherapy). In **Figure 3**, we show an upright CT scanner and the upright patient positioner and that is currently being developed by one of these companies and that will soon be available for integration into existing proton therapy systems.

#### Clinical Potential Benefits of Upright Treatments

The potential advantages of treating the patient in a seated or upright position have been addressed by several scholars in the field. Verhey et al. reported in 1981 that patients can be immobilized effectively in the seated position with less unwanted motions than in a supine or decubitus position (92). McCarroll et al. reported on the benefits of treating thoracic and Head and neck patients in a seated position (93). Yang et al. reported that thoracic patients breathe easier and are more relaxed in an upright position while the lung volume is on average up to 25% larger compared to the supine position and the excursion of a lung tumor as a result of breathing motion is also smaller (94), depending on the location of the tumor in the lung. The WHO reported recently that 55% cancer deaths are from disease sites that are affected by breathing motion (95). Treating these cases in a seated or upright position could then result in improved patient comfort, less target motion and less lung volumes being irradiated.

Among the main benefits of having the patient immobilized in an upright position we can mention (a) the reduced risk of asphyxiation and (b) the reduced need to swallow that causes significant movement in the neck and esophageal regions (93). Applying anesthesia to patients in the upright position is common practice, e.g., in shoulder and posterior fossa surgery and might also be safer in some cases with respect to the risk of asphyxiation, providing a proper support system for such patients is developed. It also depends on the airway management as well as the depth of anesthesia. Treating quadriplegic and paraplegic patients in the upright position should also be easy since it is standard practice to support such patients in upright positions for many clinical reasons.

#### Technical Benefits of Upright Treatments

Upright treatments may offer several technical benefits. Rotating a 100 to 250 Kg patient isocentrically is mechanically easier than moving a more than 50T gantry around the patient with the required precision. This of course means that one needs to focus on patient comfort and proper immobilization of the patient in the upright position. In a recent special edition of the British Journal of radiology (BJR), one of us (96) listed eight beam delivery specific technologies that proton therapy systems must be able to offer within the next ten years. Most of these technologies seems to be more attainable in a fixed beam configuration for mainly two reasons. Firstly, fixed beam arrangements provide much more free space around the isocenter compared to “closed” gantries, and the treatment envelope around the patient is much more accessible and predictable. The difference is lower with “open” gantries with partial isocentric rotation. Furthermore, the beam delivery nozzle could be retracted further to provide the required space to implement some of these technologies, e.g., on line axial CT at isocenter and proton imaging. Second, the fixed beam systems may be much less expensive, reducing the total project cost and so the barriers to their purchase.

Cone beam CT (CBCT) images of the patient could easily be obtained while rotating the patient precisely in a stationary x-ray beam measuring the transmission x-rays with stationary x-ray detectors. Proton radiography (P-Rad) and proton computed tomography (PCT) images can be obtained in a similar manner providing the proton beam energy is sufficient for the protons to traverse the specific anatomical region. Upright treatments require only fixed beam lines which will allow for moving the scanning magnet further away from the isocenter. This in turn will allow for faster beam scanning since less scanning power is required. The benefits of faster scanning are important in terms of organ motion, FLASH radiation therapy and PAT. Other benefits of moving the scanning magnet further away from the isocenter are a larger source to axis distance (SAD) and the ability to scan the beam to larger field sizes. If a fixed beam delivery nozzle is equipped with a collapsible vacuum section or a helium bag smaller spot sizes can be achieved. This will also allow for variable spots sizes since the beam control does not have to accommodate variations in the beam optics as a result of changes in the gantry angle. Implementing fast trimmer apertures would also be much easier since the gravitational forces on the trimmer components will be constant (96).

The benefits of upright treatments in reducing the cost of a proton therapy system seem self-evident. Fixed beams are cheaper to construct and much easier to maintain as they are comprised of few and mostly static components. Installing and commissioning fixed beam systems will also be faster which will result in significant project cost savings. The shielded volume for a fixed beam system is much smaller and the wall thicknesses can be reduced significantly over the bulk of the shielded volume since the primary beam will only be directed in one direction. This could allow for optimizing the treatment room layout resulting in significant cost reductions. The latter could also allow for improved treatment workflow and throughput efficiencies. The traditional clinical concerns around upright treatments could be outweighed by the potential benefits that upright treatments hold for many patients.

## Discussion and Conclusions: The Link Between Biology and Mechanics

In spite of more than 50 years of application of protons, this is still a highly evolving branch of radiation therapy. It is synergic with the developments with photon and ion beams. A multidisciplinary and multicentric approach is necessary to advance in this field, as it is true for all the tools in the treatment of cancer.

**Figure 4:**
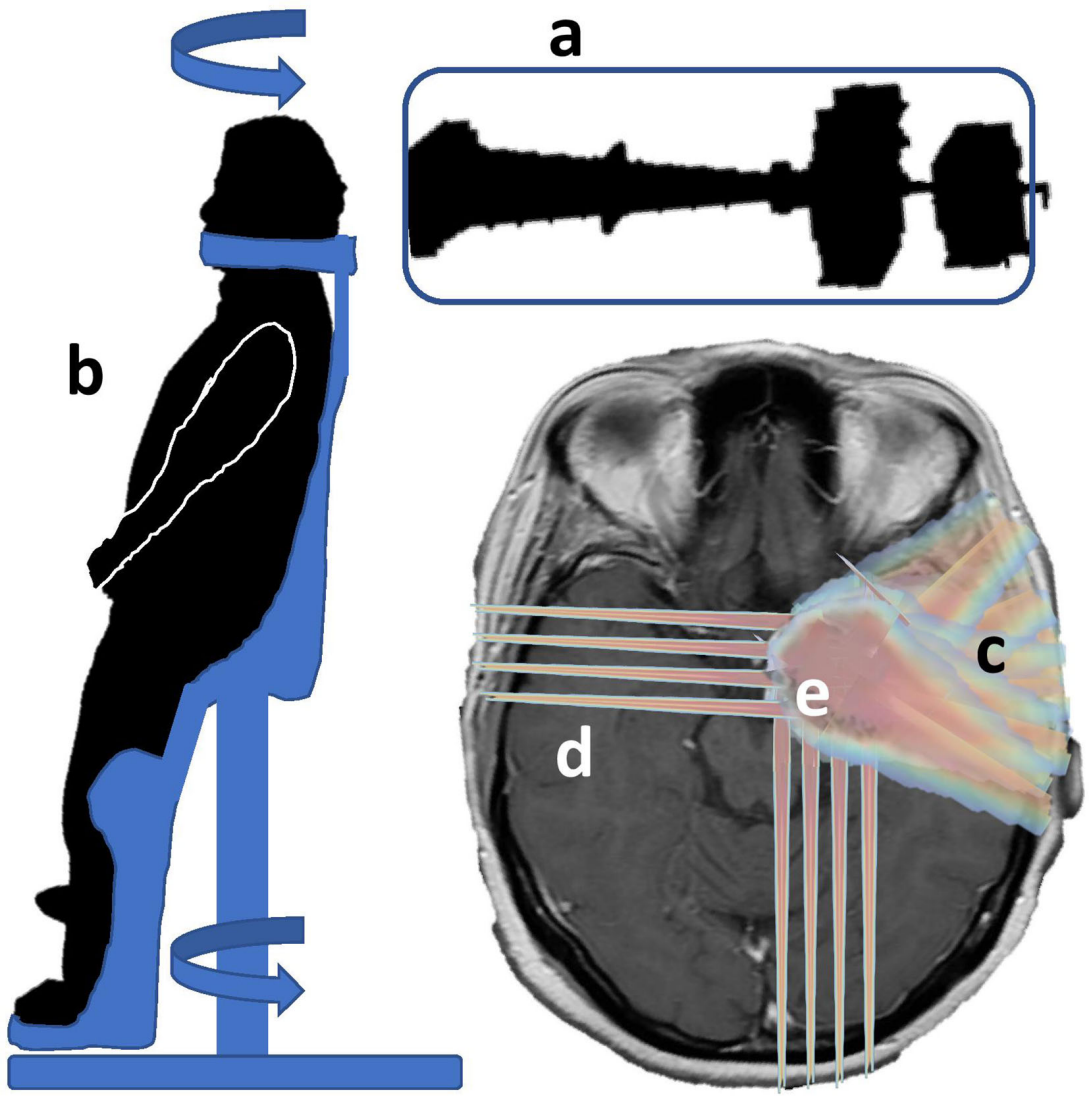
Combination of biological and technical aspects to reduce pitfalls in proton therapy: the combination of a fixed line “a” with a rotational patient positioner “b” could reduce the investment cost. It is compatible with arc therapy, maybe for partial arcs “c” in order to avoid a large spread of low doses and keeping the original rationale for protons. Mini-beams could be applied for beam incidences traversing a large amount of healthy tissue “d” and, synergically with FLASH, be applied in specific regions “e” of the tumor and healthy tissues, protecting specific regions or organs.

In this work, we have reviewed aspects that can individually reduce some of the pitfalls of proton therapy. Even if they seem to be disconnected (biology and mechanics), some synergies or incompatibilities can be found between them based on the described process for each, as represented in **Figure 4**.

FLASH can reduce the damage to normal tissues under specific conditions including beam parameters (minimal dose, maximal time) and oxygenation. Different studies have shown that with the present devices it is difficult to achieve the technical conditions for FLASH (85) and even more in large volumes. In the short and mid-term it can be foreseen that FLASH will apply to treating smaller volumes close to or embedded in the target volume if the differential effect of FLASH, between tumoral and healthy cells, is not only related to oxygen, but also to cellular factors. While this scenario is the usual one in radiation therapy, specific situations should also be studied. One example could be re-irradiation in or close to critical organs, or in vascular areas, to cumulated doses of 110 to 140 Gy, where the risk of necrosis or injuries to vessels are high and with very different levels of oxygenation.

Minibeams could be applied in synergy with FLASH in order to avoid any movement, and optimized for large paths through healthy tissue and applied to small target volumes differentiating the benefit between tumoral and normal cells or, more specifically, organs [eg hippocampus, (14)].

In contrast, proton arc therapy is in principle not easily compatible with minibeams, and can also affect some mechanisms on the immune response to radiation therapy if large volumes are irradiated again with low doses.

It is important to conclude mentioning that even among the co-authors of this review, where we also include personal work of some of them, there is not a unanimous agreement on the potential effect of the proposed scenarios, interpretations and tools. It is not yet known how many logistical and flexible advantages will be lost without a gantry, how much the pattern of dose distribution with rotational techniques will change the response of tissues, how really mini-beams should be delivered to keep the tumor control with inhomogeneous dose, and where, why and how FLASH will be applied efficiently.

If we succeed, with one of these approaches, to reduce at least one or some of the pitfalls of proton therapy in its present status (such as cost, complexity, downtime, uncertainties and complications), it will be even easier to find a better place of protons as a therapy of choice for treating cancer with radiation therapy, in a multidisciplinary approach, for a wider population.

## Author Contributions

AM: manuscript design and figures. JV: dosimetry section. DS-P: arc therapy. JU, SE, VS-T, LF: time signal data. PB, AG-U: minibeams and FLASH. NG, GG: physics review. JC, JP: medical physics review. LM, AI, CA, RM: clinical review. MC: anesthesia review. NS: gantryless section. All: literature review. All authors contributed to the article and approved the submitted version.

## Funding

This work was partially funded by Comunidad de Madrid (Project B2017/BMD-3888 PRONTO-CM “Proton therapy and nuclear techniques for oncology” and project 2017-T1/BMD-5468), Spanish Government (RTI2018-098868-B-I00, RTC-2015-3772-1, PID2019-104991RB-I00), European Regional Funds, EU Marie Sklodowska-Curie program (grant agreement 793576-CAPPERAM) is acknowledged. This is a contribution for the Moncloa Campus of International Excellence, “Grupo de Física Nuclear-UCM,” Ref. 910059.

## Conflict of Interest

NS is the President of proton therapy for Leo Cancer Care, Ltd.

The remaining authors declare that the research was conducted in the absence of any commercial or financial relationships that could be construed as a potential conflict of interest.
